# Collapse risk assessment based on linear programming variable weight-cloud model

**DOI:** 10.1371/journal.pone.0311951

**Published:** 2024-12-16

**Authors:** Xiaoyi Zhou, Ke Hu, Tingqiang Zhou

**Affiliations:** Department of Civil Engineering, Chongqing Three Gorges University, Wanzhou, Chongqing, China; Zhejiang A&F University, CHINA

## Abstract

Collapse risk assessment is an important basis for the prevention and control of geological disasters in mountainous areas. The existing research on collapse hazard is less, and there is still no further advancement in the evaluation of collapse hazard for the traditional indicator assignment method for the diversification of the assignment results of the indicators and the comprehensive evaluation method that cannot consider the ambiguity and randomness of the indicator data at the same time. In this paper, we utilize the respective advantages of the linear programming theory and the cloud model from the prevention and control point of view, and evaluate the collapse samples. Firstly, the weight interval of evaluation index is determined by improved analytic hierarchy process, entropy weight method and coefficient of variation method. Secondly, the linear programming algorithm is used to select the specific weight of each collapse sample when the risk is the largest in the interval. Finally, a comprehensive evaluation model of cloud model is constructed to determine the risk level of collapse. In this paper, 20 collapse samples counted by predecessors in G4217 Wenchuan-Lixian section are taken as research cases. The evaluation results of 20 collapse samples are compared with other evaluation methods and field survey conditions to prove the reliability and rationality of the method. The evaluation results show that 13 of the 20 collapse samples are extremely dangerous, 2 are highly dangerous, 4 are moderately dangerous, and 1 is lowly dangerous. Among them, the extremely dangerous collapse samples account for 65% of the total number of collapses. Compared with other methods, this method is more in line with the actual situation.

## 1 Introduction

In the western region of China, due to the complex terrain and geological conditions, fragile ecological environment and frequent seismic activity, the frequency of collapse is very high. These disasters have a great negative impact on people ’s production and life and the country ’s economic development [1, 2]. Therefore, carrying out collapse risk assessment plays an important role in preventing collapse disasters [3, 4].

At present, most of the collapses in mountainous areas are rockfalls or tunnel collapses. These geological disasters are sudden, uncontrollable, destructive and difficult to predict [5]. Because of the many influencing factors, and the existing research on the collapse risk is relatively small, which makes the evaluation of the collapse risk is one of the difficulties of engineering geology research [6–9]. At present, the form about the evaluation of collapse risk is mainly based on the establishment of evaluation index system, combined with fuzzy comprehensive evaluation method, for example: Jin et al. [10] first established a mathematical model based on a large number of collapse disaster survey data and socio-economic statistical information to evaluate the risk of collapse disasters. Chen et al. [11] used DS evidence theory and fuzzy comprehensive evaluation method to construct a risk assessment model of collapse geological disasters. Wang et al. [12] used the analytic hierarchy process and correlation analysis to determine the subjective weight and objective weight of the evaluation index when evaluating the collapse disaster of mountain tunnels, and evaluated the tunnel collapse in combination with the mountain tunnel collapse risk assessment system. Liu et al. [13] carried out the risk assessment of geological disasters in the study area by using GIS system and analytic hierarchy process. Xiao et al. [14] calculated the weight of evaluation index by G1 method, and established the risk assessment model of a highway collapse in China based on the spatial analysis function and factor superposition method of ArcGIS. Xu et al. [15] used a combination of qualitative and quantitative methods to describe the indicators, used the frequency statistics method to calculate the weight of the evaluation indicators, established the attribute measure function to calculate the single index and the comprehensive attribute measure, and finally used the confidence criterion to determine the risk level of loess tunnel collapse. Guerriero et al. [16] used GIS combined with analytic hierarchy process to establish a multi-disaster risk assessment model, which provided a method reference for multi-disaster assessment. He et al. [17] selected 15 evaluation indexes by considering the characteristics of the study area and combined with the theory of unconfirmed measurement to evaluate the collapse hazard. In summary, the important research results of scholars at home and abroad have laid a theoretical foundation for the hazard evaluation of collapse disasters, but there are still some shortcomings, for example: (1) There are deficiencies in the weight assignment methods of indicators applied to the evaluation of collapse risk. Although there have been studies on combining qualitative and quantitative evaluation in the evaluation of collapse risk, different weighting methods often give different weighting results to the evaluation indicators, which leads to a large deviation in the evaluation results after the combination of weighting, and at the same time, the common combination of weighting methods is difficult to provide a comprehensive weighting evaluation results, and it is difficult to consider and combine the fuzzy nature of the weighting results. (2) Most of the existing methods of evaluation by fuzzy mathematical methods in the evaluation of collapse risk have poor applicability and reliability. In different areas, the changes of indicator assignment methods and evaluation indicators will affect the calculation of the affiliation degree of the comprehensive evaluation method, and the whole calculation process is more complicated, which is not able to present the evaluation results more intuitively. Therefore, in addition to the need for reasonable indicator weights, the selection of a comprehensive evaluation method with strong reliability, good applicability and a simple calculation process is also a key process.

Based on the above deficiencies, this paper proposes a method for evaluating the collapse risk based on the linear programming variable weight-cloud model. Firstly, the Improved Analytic Hierarchy Process (IAHP), Entropy Weight method (EW) and Coefficient of Variation method (CV) are used to calculate the initial weights. Compared with the traditional hierarchical analysis method, IAHP simplifies the consistency test process, and the subjective weights of the indexes can be calculated in a simpler way, and the two methods of EW and CV are more reliant on the indexes’ data, and the objective weights of the indexes can be calculated, and the fusion of the three methods can obtain more comprehensive weight evaluation results. The integration of the three methods can obtain a more comprehensive weight evaluation result. Secondly, a fuzzy weight interval is established using the weights calculated by the above method, the above interval is used as a constraint and the maximum hazard score is used as an objective function, based on the objective function and the constraints, the final weights of the indicators are calculated using linear programming. Finally, the comprehensive evaluation method chosen in this paper is the cloud model, which has strong applicability and reliability in dealing with the problem of multi-indicator and multi-criteria, and can transform the fuzzy and stochastic hazard level into quantitative certainty, so as to reveal the uncertainty law in the evaluation of hazards, and validate the feasibility of the method by comparing it with other evaluation methods.

In section 2, the theory related to constructing the integrated evaluation method of linear programming variable-weight-cloud model in this paper is introduced; in section 3, the process of constructing the model as well as the establishment of the index system are introduced; in section 4, an example is utilized to validate the model and the evaluation results are analyzed; and the reasonableness as well as the limitations of the model are discussed in section 5.

## 2 Methods

Linear Programming Variable Weighting is to use linear programming theory as a method of changing the weights of the indicators, using the results calculated by multiple weighting methods as a weight interval, and combining each sample data to solve the optimal weights of each sample indicator. In order to enhance the diversity of index weights, this paper selects the improved analytic hierarchy process, entropy weight method and coefficient of variation as the method to determine the weight interval.

### 2.1 The method for determining the weights

#### 2.1.1 Improved analytic hierarchy process

The improved analytic hierarchy process uses the three-scale method to construct a pairwise comparison judgment matrix. Compared with the 9-scale of the traditional analytic hierarchy process, the improved analytic hierarchy process is easier to judge the importance between the indicators. Eliminate the blindness of people in constructing the hierarchical judgment matrix, while omitting the consistency test process and enhancing the scientific nature of decision-making. The specific improvement process [18] is as follows:

(1) Since the “1–9” scale in the traditional AHP requires high accuracy in judging the degree of importance, and it is difficult to eliminate people’s subjective views. Therefore, the Improved Analytical Hierarchy Process (IAHP) adopts the “0–2” scale instead of the “1–9” scale.
(2) The comparison matrix constructed by the traditional AHP through expert evaluation, considering that even the same model may have different effects in different projects, this paper adopts the “backward projection method” to construct the comparison matrix; firstly, a single model is used to make a prediction, and then the comparison matrix is constructed according to the prediction results [18].
(3) The improved hierarchical analysis method introduces the concept of optimal transfer matrix in the literature [19], which avoids the practice of repeatedly checking the consistency in the traditional AHP, reduces the amount of calculation and simplifies the model.

The specific calculation steps of the improved AHP are as follows:

(1) Establish the hierarchical structure model. The upper layer is the target layer, the middle is the index layer, and the lower layer is the program layer.
(2) Construct the comparison matrix using the “0–2” three-scale method. First of all, the relative importance of n factors, after expert scoring, to establish the initial judgment matrix A:

$$A={\left(a_{ij}\right)}_{n\times n}={\left(\begin{array}{ccc} a_{11} & \cdots & a_{1j}\\ \vdots & \ddots & \vdots \\ a_{i1} & \cdots & a_{ij} \end{array}\right)}_{n\times n}$$ (1)

In the formula, *a*_*ij*_ denotes the importance of indicator *i* in relation to indicator *j*; *i* = 1,2, 3, …, n; *j* = 1, 2, 3, …, *n*; *n* is the order of the matrix. *a*_*ij*_ is as follows:

$$a_{ij}=\left\{\begin{array}{l} \begin{array}{cc} 0, & \text{the}\;i\;\text{factor}\;\text{is}\;\text{not}\;\text{as}\;\text{important}\;\text{as}\;\text{the}\;j\;\text{factor} \end{array}\\ \begin{array}{cc} 1, & \text{the}\;i\;\text{factor}\;\text{is}\;\text{as}\;\text{important}\;\text{as}\;\text{the}\;j\;\text{factor} \end{array}\\ \begin{array}{cc} 2, & \text{the}\;i\;\text{factor}\;\text{is}\;\text{more}\;\text{important}\;\text{as}\;\text{the}\;j\;\text{factor} \end{array} \end{array}\right.$$ (2)

According to the judgment matrix, A is established, and the importance ranking index of each factor is calculated:

$$r_{i}=\sum_{j=1}^{n}a_{ij},i=1,2,3,\cdots ,n$$ (3)

In the formula, *r*_*i*_ is the sum of the elements in the ith row of matrix A.

(3) Construct the judgment matrix B, the element *b*_*ij*_ should meet the following conditions:

$$b_{ij}=\left\{\begin{array}{l} \frac{r_{i}-r_{j}}{r_{\max}-r_{\min}}\left(k_{m}-1\right)+1,r_{i}-r_{j}\geq 0\\ 1/\left|\frac{r_{j}-r_{i}}{r_{\max}-r_{\min}}\left(k_{m}-1\right)+1\right|,r_{i}-r_{j}<0 \end{array}\right.$$ (4)

In the formula, *b*_*ij*_ denotes the value of the ith row and jth column in the judgment matrix B. *r*_*max*_ = max{*r*_*i*_}, denotes the element corresponding to the maximum sorting index; *r*_*min*_ = min{*r*_*i*_}, denotes the element corresponding to the minimum sorting index; and *k*_*m*_ *= r*_*max*_*/r*_*min*_, denotes the degree of significance given by a certain degree when *r*_*max*_ and *r*_*min*_ are compared.

(4) Find the optimal transfer matrix C:

$$c_{ij}=\frac{1}{n}\sum_{k=1}^{n}\left(\text{lg}\frac{b_{ik}}{b_{jk}}\right)$$ (5)

In the formula, *c*_*ij*_ is the value of the *ith* row and *jth* column of the transfer matrix C, *n* is the order of the matrix, *k* = 1,2, 3, …, *n*.

(5) Finding quasi-optimal consistent matrix D:

$$d_{ij}=10^{c_{ij}}$$ (6)

In the formula, *d*_*ij*_ is the value of the *ith* row and *jth* column of the fitted consistency matrix D.

(6) Calculate the relative weight *ω*_*i*_ of each level index factor:

$$\omega_{i}=\frac{\bar{\omega_{i}}}{\sum_{i=1}^{n}\bar{\omega_{i}}}$$ (7)

The ${\bar{\omega }}_{i}$ in the above equation is shown below:

$${\bar{\omega }}_{i}=\sqrt[{n}]{\prod_{j=1}^{n}d_{ij}}$$ (8)

The single-layer weight vector *ω* = (*ω*_1_,*ω*_2_,*ω*_3_,⋯,*ω*_*n*_) is obtained.

(7) Calculate the combined weight *V*:

$$V_{i}=\sum_{k=1}^{n}\omega_{k}•\omega_{ik}$$ (9)

In the formula, *V*_*i*_ denotes the final weight of the *ith* indicator, *ω*_*k*_ is the factor weight corresponding to the upper level of the hierarchy in which *ω*_*ik*_ is located, and *ω*_*ik*_ is the weight corresponding to the *ith* indicator that is in the *kth* factor.

#### 2.1.2 Entropy weight method

The entropy weight method is a method to measure the importance of evaluation indicators based on the size of information entropy [20]. When the information entropy value is smaller, the greater the degree of dispersion of the evaluation index, the greater the impact on the comprehensive evaluation object. If the values of a certain index are all the same, the index is invalid in the comprehensive evaluation. In the collapse risk assessment, the specific steps of applying the entropy weight method are as follows:

(1) The dimensionless treatment of *x*_*ij*_ is carried out to obtain $x_{ij}^{\prime }$:

Positive indexes:

$$x_{ij}^{'}=\frac{x_{ij}-\min(x_{ij})}{\max(x_{ij})-\min(x_{ij})}+\alpha$$ (10a)

Reverse Indicators:

$$x_{ij}^{'}=\frac{\min(x_{ij})-x_{ij}}{\max(x_{ij})-\min(x_{ij})}+\alpha$$ (10b)

In the formula, *x*_*ij*_ is the value of the *jth* indicator of the *ith* sample, *min*(*x*_*ij*_) is the minimum value, *max*(*x*_*ij*_) is the maximum value, to eliminate the effect of the 0-value, a very small value *α* is added to the dimensionless processed $x_{ij}^{\prime }$, In this paper *α* is taken to be 0.001.

(2) Standardized processing, the proportion of the *jth* index of the *ith* sample of *P*_*ij*_ in the overall data:

$$P_{ij}=\frac{x_{ij}^{*}}{\sum_{i=1}^{n}x_{ij}^{*}}$$ (11)

(3) Calculate the difference coefficient *g*_*i*_ of the *jth* index:

$$g_{i}=1+\frac{1}{\text{ln}\;n}\sum_{i=1}^{n}p_{ij}\;\text{ln}\;p_{ij}$$ (12)

(4) Calculate the weight *ω*_*j*_ of the *jth* index:

$$\omega_{j}=\frac{g_{i}}{\sum_{j=1}^{m}g_{i}}$$ (13)

#### 2.1.3 Coefficient of variation method

The coefficient of variation method [21] calculates the coefficient of variation by solving the mean and standard deviation of the sample data, and normalizes the coefficient of variation as the index weight and is used for comprehensive evaluation. The calculation process is as follows:

(1) Assuming that there are n samples and p evaluation indexes, the original index numerical matrix is constructed:

$$X=\left(\begin{array}{ccc} x_{11} & \cdots & x_{1p}\\ \vdots & \ddots & \vdots \\ x_{n1} & \cdots & x_{np} \end{array}\right)$$ (14)

Where, *x*_*ij*_ represents the value of the *jth* evaluation index of the *ith* sample.

(2) Calculate the mean and standard deviation of the *jth* indicator:

$$\left\{\begin{array}{c} \bar{x_{j}}=\frac{1}{n}\sum_{i=1}^{n}x_{ij}\\ S_{j}=\sqrt{\frac{\sum_{i=1}^{n}{\left(x_{ij}-\bar{x}\right)}^{2}}{n-1}} \end{array}\right.$$ (15)

(3) Calculate the coefficient of variation of the *jth* index:

$$V_{j}=\frac{S_{j}}{x_{j}},j=1,2,3,\cdots ,p$$ (16)

(4) The coefficient of variation of each index is normalized to obtain the weight of each evaluation index *ω*_*j*_:

$$\omega_{j}=\frac{V_{j}}{\sum_{j=1}^{p}V_{j}}$$ (17)

### 2.2 Axial strain method

**Fig 1:**
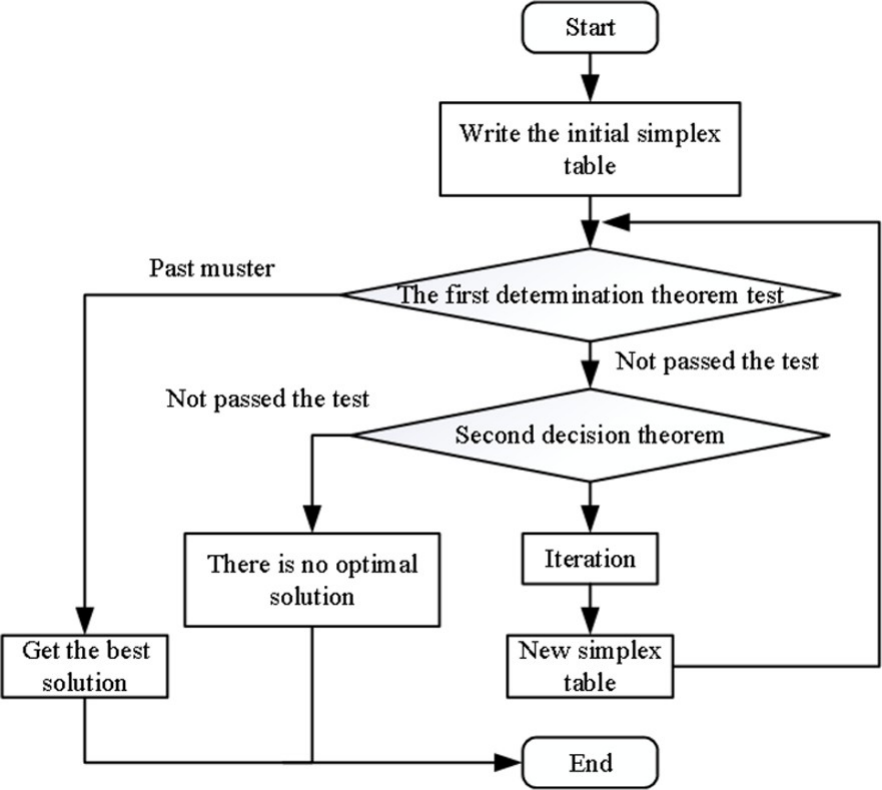
Flow chart of linear programming.

Linear programming [22] is an important branch of operations research. It was first applied to military, economic, management and other disciplines. It is generally composed of decision variables, constraints and objective functions. When applying linear programming theory to solve the optimal solution or objective function value, we must first determine the decision variables, then find the constraint conditions that meet the decision variables (that is, find the linear equality or inequality constraints), and finally find the extremum of the objective function within the constraint conditions. The linear programming problem can be divided into single-objective linear programming and multi-objective linear programming according to the objective function. According to the characteristics of collapse risk assessment, this paper considers only solving a maximum risk value, and uses single-objective linear programming to solve the index weight and risk score corresponding to each collapse sample. The basic idea of single-objective linear programming is shown in Fig 1.

The main steps applied in this paper are:

(1) In order to avoid the influence of the evaluation index dimension, we should first apply Eq (10) to the dimensionless processing of each evaluation index, map the sample data to [0,1], and regard the dimensionless data of each collapse sample as the coefficient corresponding to each decision variable in the objective function.
(2) In the collapse risk assessment, the objective function is the risk score *Z*_*i*_(*x*_*j*_) corresponding to each collapse sample, which is recorded as *Z*_*i*_(*u*_*j*_) *= x*_1_*ω*_1_*+x*_2_*ω*_2_*+···+x*_*j*_*ω*_*j*_, where *x*_*j*_ is the normalized value of the evaluation index, *ω*_*j*_ is the weight of each corresponding index (decision variable), *i* is the *ith* collapse sample, and *j* is the *jth* evaluation index.
(3) The weight interval is constructed by using the index weight results calculated by each weight method. At this time, the weight interval is the constraint condition of the linear programming objective function.

$$\left\{\begin{array}{l} \omega_{1}\in \left[\omega_{1\min},\omega_{1\max}\right],\omega_{2}\in \left[\omega_{2\min},\omega_{2\max}\right]\\ \omega_{3}\in \left[\omega_{3\min},\omega_{3\max}\right],\omega_{4}\in \left[\omega_{4\min},\omega_{4\max}\right]\\ \omega_{5}\in \left[\omega_{5\min},\omega_{5\max}\right],\omega_{6}\in \left[\omega_{6\min},\omega_{6\max}\right]\\ \omega_{7}\in \left[\omega_{7\min},\omega_{7\max}\right],\omega_{8}\in \left[\omega_{8\min},\omega_{8\max}\right]\\ \omega_{9}\in \left[\omega_{9\min},\omega_{9\max}\right],\omega_{10}\in \left[\omega_{10\min},\omega_{10\max}\right]\\ \omega_{11}\in \left[\omega_{11\min},\omega_{11\max}\right],\omega_{12}\in \left[\omega_{12\min},\omega_{12\max}\right] \end{array}\right.$$ (18)

**Fig 2:**
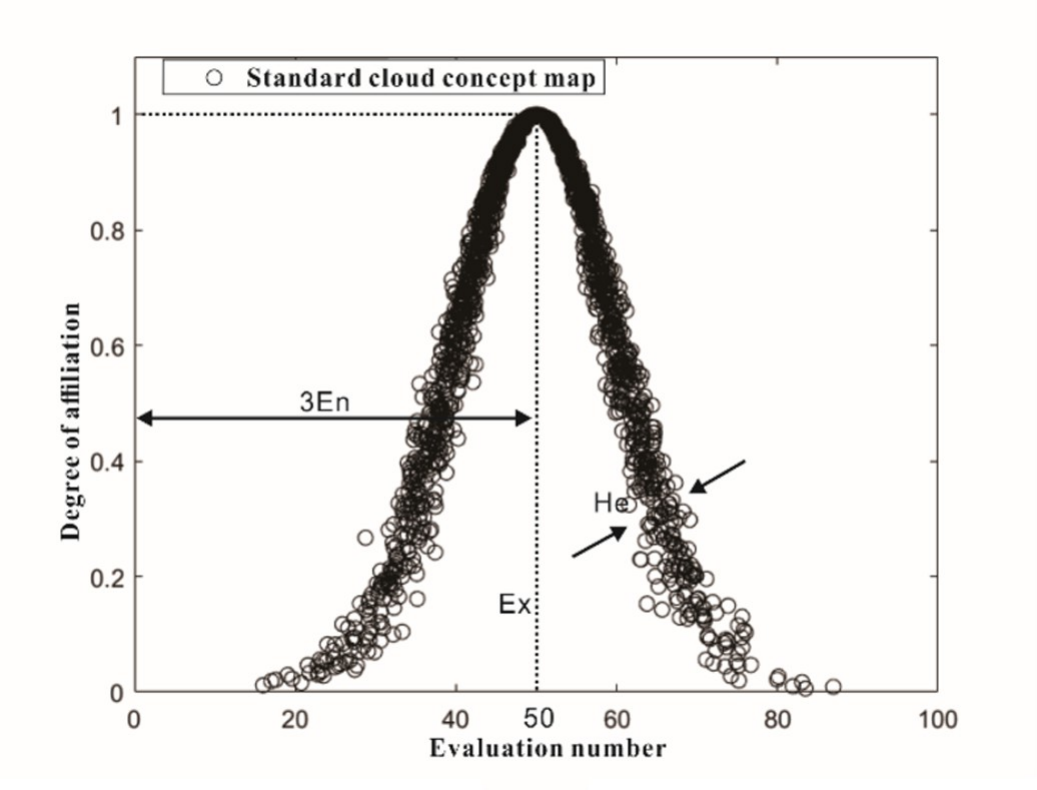
Concept map of cloud model.

(4) The standard form of linear programming can be established by using the objective function and weight interval determined by steps (2) and (3).
(5) According to the process shown in Fig 2, the initial basic feasible solution *ω*(0) is found, and whether to enter the iteration is determined according to the judgment conditions. If *ω*(0) does not meet the conditions, it is regarded as the starting value of the iterative process to find the feasible solution *ω*(1), so that the objective function value *z*(*ω*(1)) ≤ z(*ω*(0)).
(6) If *ω*(1) does not meet the conditions, continue to find *ω*(2), *ω*(3), *ω*(4), and improve the objective function according to the judgment conditions. The optimal weight solution and the objective function value are obtained at the end of the iterative process.

### 2.3 Cloud model theory

Cloud model [23] is a cognitive model proposed by academician Li Deyi, which is used to transform qualitative knowledge and quantitative knowledge. The model has been widely used in data mining, intelligent control, surrounding rock stability classification, rock burst classification and other fields.

(1) The definition and digital characteristics of cloud. The cloud model controls the shape of the model through three digital features (expected Ex, entropy En, hyper entropy He), and generates a large number of cloud droplets to form a cloud droplet group through the cloud generator, thereby realizing the uncertainty conversion between the qualitative concept and its quantitative representation. The definition of cloud is as follows:

Let Z be a quantitative interval of a qualitative concept. If *x*∈*Z*, *x* denotes a random realization of a qualitative concept in *Z*. Every *x* in *Z* has a stable tendency of membership *u*(*x*)∈[0,1]. Then all the cloud droplets distributed on *Z* are called clouds. The standard cloud model diagram is shown in Fig 2.

The three numerical characteristics of the cloud control the range and shape of the cloud, where Ex is expected to reflect the center of gravity of the cloud droplets; the entropy En reflects the degree of dispersion and ambiguity of the concept extension. The super-entropy He represents a re-description of fuzziness.

(2) Cloud model implementation.
① Determine the evaluation level standard cloud:

According to the grade standard of evaluation index, Eq (19) is used to calculate the standard cloud digital features.


$$\left\{\begin{array}{l} Ex_{v}=\left(C_{v}^{\max}+C_{v}^{\max}\right)/2\\ En_{v}=\left(C_{v}^{\max}-C_{v}^{\max}\right)/6\\ He_{v}=kEn_{v} \end{array}\right.$$ (19)

In Eq (19), $C_{V}^{max}$, $C_{V}^{min}$ are the upper and lower boundaries of the *V* grade interval, *k* is a constant, here take 0.1.

② Determine the evaluation cloud:

According to the basic data of the collapse sample, the three digital features of the evaluation cloud are calculated according to Eq (20).


$$\left\{\begin{array}{l} E_{xj}=\sum_{i=1}^{n}x_{ij}/n\\ E_{nj}=\sqrt{\frac{\pi }{2}}\left(\sum_{i=1}^{n}\left|x_{ij}-E_{xj}\right|/n\right)\\ H_{ej}=\sqrt{\left|S_{j}^{2}-E_{nj}^{2}\right|}\\ S_{j}^{2}=\sum_{i=1}^{n}{\left(x_{ij}-E_{xj}\right)}^{2}/\left(n-1\right) \end{array}\right.$$ (20)

Where *n* is the number of samples, *m* is the number of indicators; *x*_*ij*_ (*i* = 1, 2, …, *n*; *j* = 1, 2, …, *m*) is the data of the *jth* evaluation index of the *ith* sample; $S_{j}^{2}$ is the sample variance.

③ Determine the comprehensive evaluation cloud:

The comprehensive evaluation cloud is based on the cloud model fusion algorithm, which combines the specific index weight *ω*_*j*_ with the evaluation cloud, as shown in Eq (21).


$$\left\{\begin{array}{l} E_{x}=\sum_{j=1}^{m}E_{xj}*\omega_{j}\\ E_{n}=\sqrt{\sum_{j=1}^{m}E_{nj}*\omega_{j}}\\ H_{e}=\sum_{j=1}^{m}H_{ej}*\omega_{j}^{2} \end{array}\right.$$ (21)

④ Determine the comprehensive level risk cloud:

Since the weight of this paper is planned according to each sample, in order to ensure the consistency of the comprehensive evaluation standard cloud, the risk level comprehensive cloud is constructed as Eq (22).


$$\left\{\begin{array}{l} E_{xV}=\sum_{j=1}^{m}E_{xv}*\bar{\omega_{j}}\\ E_{nV}=\sqrt{\sum_{j=1}^{m}E_{nv}*\bar{\omega_{j}}}\\ H_{eV}=\sum_{j=1}^{m}H_{ev}*\bar{\omega_{j}} \end{array}\right.$$ (22)

In the formula, $\bar{\omega_{j}}=\sum_{i=1}^{n}\omega_{ij}/n$ represents the weight expectation of the *jth* evaluation index.

⑤ Determine risk grade:

According to the numerical characteristics of the comprehensive evaluation cloud and the comprehensive grade risk cloud, the two are drawn in the same spatial coordinate system to determine the risk level. The standard cloud closest to the comprehensive cloud is the final evaluation result.

## 3 Model construction

### 3.1 Basic ideas

**Fig 3:**
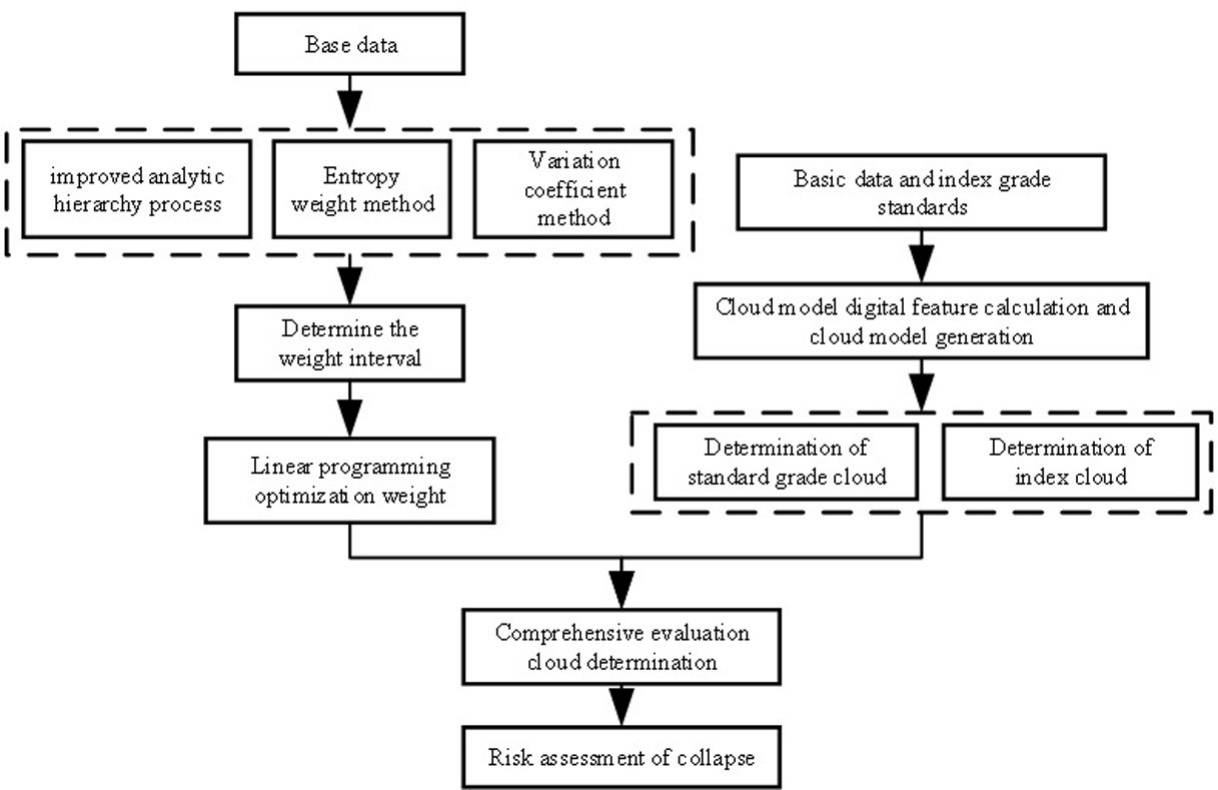
The overall flow chart.

The overall flow chart of the model calculation is shown in Fig 3:

(1) The subjective weight and objective weight of each index are calculated by improved analytic hierarchy process, entropy weight method and coefficient of variation method to determine the constraint interval of linear programming algorithm.
(2) According to the determined constraint conditions, the linear programming standard model is constructed, and the linear programming algorithm is used to solve the optimal solution of the index weight of each collapse sample according to the most dangerous score.
(3) Calculate the digital characteristics of the standard cloud according to the index grade standard, and calculate the digital characteristics of each index cloud according to the sample basic data.
(4) Based on the calculated index cloud digital features, standard cloud digital features, and sample index weights, the comprehensive cloud digital features are calculated, the comprehensive cloud model is generated, and the collapse risk level is determined.

### 3.2 Index system construction and grade standard determination

**Fig 4:**
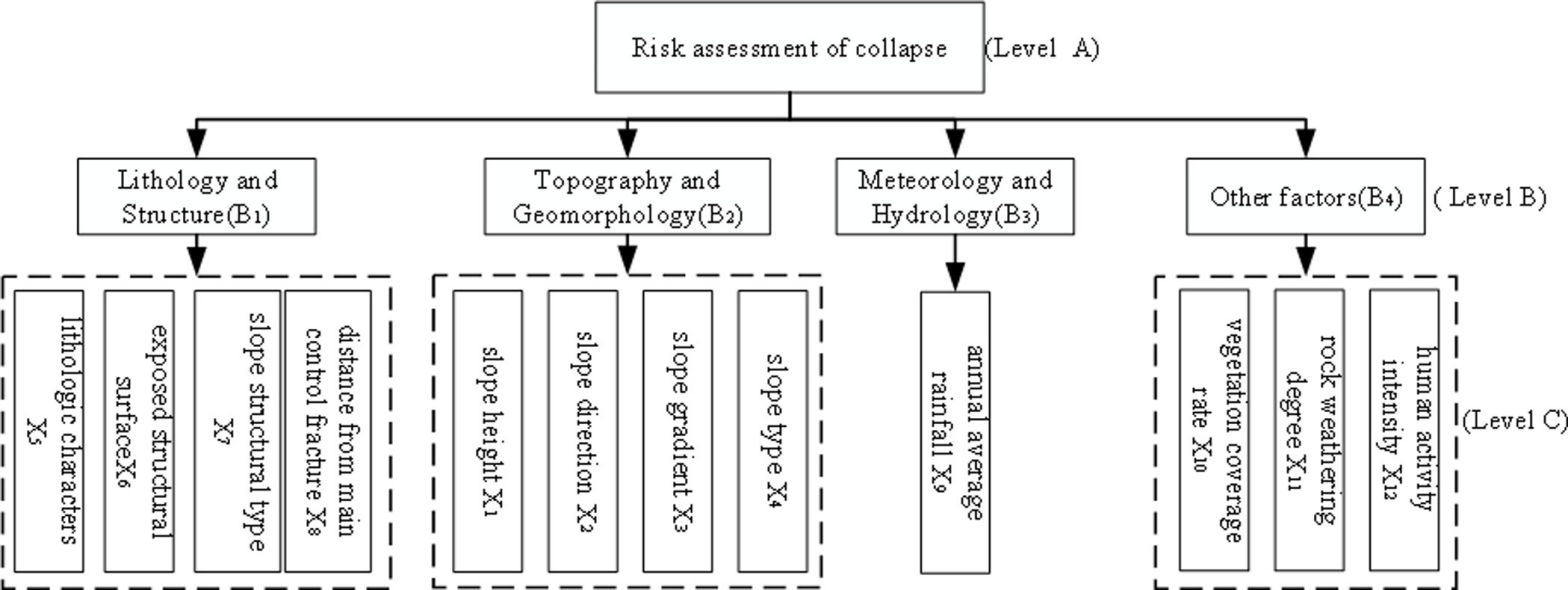
Collapse risk evaluation index system.

Scientific and reasonable selection of evaluation indexes is very important for accurately predicting the risk level of collapse disasters. Because there are many factors affecting the collapse disaster, considering the limitations of various objective conditions, it is impossible to reflect all the evaluation indexes into the collapse risk assessment. Therefore, the selected evaluation indexes should have clear representativeness and clear physical meaning, and each evaluation index is independent of each other and easy to quantify. This paper comprehensively considers the research results of predecessors in this field [24–27] and selects 12 factors such as slope height (X_1_), slope direction (X_2_), slope gradient (X_3_), slope type (X_4_), lithologic characters (X_5_), exposed structural surface (X_6_), slope structural type (X_7_), distance from the main control fracture (X_8_), annual average rainfall (X_9_), vegetation coverage rate (X_10_), rock weathering degree (X_11_), human activity intensity (X_12_) as evaluation indexes. The level of collapse risk evaluation index is shown in Fig 4. Referring to the index grading standards of relevant domestic scholars [17, 24, 27], the single factor grading standard and qualitative index assignment of collapse risk assessment are determined as shown in Table 1.

**Table 1:** Evaluation indicators and grading standards.

Risk level				
Evaluating indicator	Extreme Danger(Ⅰ)	High Danger(Ⅱ)	Medium Danger(Ⅲ)	Low Danger(Ⅳ)
X_1_(m)	>200	150∼200	100∼150	<100
X_2_(°)	sunny slope (135∼225) (4)	semi-shaded slope (45∼135) (3)	semi-sunny slope (225∼315) (2)	shady slope (315∼360U0∼45) (1)
X_3_(°)	>55	45∼55	35∼45	<35
X_4_	broken-line slope (4)	concave slope (3)	linear slope (2)	convex slope (1)
X_5_	Soft loose rock (4)	soft rock (3)	soft and hard rock (2)	hard rock (1)
X_6_(team)	>3	2∼3	1∼2	0∼1
X_7_	consequent slope (4)	oblique slope (3)	cross-slope (2)	reverse Slope (1)
X_8_	0∼20	20∼40	40∼60	>60
X_9_(mm)	>500	300∼500	100∼300	<100
X_10_(%)	poorer (<10)	poor (10∼30)	good (30∼50)	better (>50)
X_11_	strongly weathered (4)	moderately weathered (3)	slightly weathered (2)	not weathered (1)
X_12_	larger (4)	large (3)	small (2)	smaller (1)

## 4 Example verification

The study area (G4217 Wenchuan-Lixian section, 56km in length) is located in the northwest side of the Longmenshan central fault zone. The terrain is undulating, the deep erosion of the river is strong, and the topographic and geological conditions are complex. Affected by the Wenchuan earthquake, the shattered rock mass on both sides of the highway is developed, and the rock mass is cut strongly in a slab shape, which makes the collapse disasters along the highway occur frequently. In this paper, 20 typical collapse points in the study area are selected as examples to verify the model [27] (see Table 2 for basic data).

**Table 2:** Sample data table of collapse risk assessment.

Sample number	Evaluating indicator											
	X_1_(m)	X_2_(°)	X_3_(°)	X_4_	X_5_	X_6_(team)	X_7_	X_8_	X_9_(mm)	X_10_(%)	X_11_	X_12_
#01	137	1	46.4	4	3	2	3	5.15	528.7	35	3	3
#02	176	4	49.3	4	3	2	4	5.9	528.7	27	3	3
#03	35	3	32.6	4	4	0	4	9.48	528.7	35	4	3
#04	351	4	58.5	4	3	3	3	9.86	528.7	8	2	4
#05	383	3	59	4	3	2	3	10.44	528.7	20	2	4
#06	191	1	64	4	4	1	3	11.89	528.7	55	4	3
#07	249	4	44.3	4	4	3	3	13.74	386.7	5	4	3
#08	226	4	52.4	4	4	0	3	14.5	386.7	5	4	3
#09	240	4	47	4	4	0	3	18.93	423	8	4	3
#10	152	4	42	4	4	0	1	18.34	423	5	4	3
#11	124	4	40	4	3	2	1	18.73	423	5	4	4
#12	189	4	45	4	3	2	1	15.8	386.7	5	4	3
#13	150	1	54.2	4	2	3	1	16.38	423	20	3	3
#14	158	1	45.3	2	2	0	1	28.65	486.5	45	4	3
#15	143	1	46	4	2	1	2	29.27	486.5	37	4	3
#16	35	1	50	2	1	2	4	32.61	486.5	25	3	3
#17	100	2	49	2	3	1	3	35.16	513.4	45	4	3
#18	115	2	53.8	4	1	0	3	37.57	513.4	5	4	3
#19	437	2	44.9	4	4	0	3	38.54	513.4	40	4	3
#20	308	1	54	4	4	0	3	42.18	650	55	4	3

### 4.1 Determine the weight interval

The weight interval in this paper is determined by the 3 methods in section 2.1, which combines the advantages of subjective methods and objective methods to comprehensively determine the weight interval.

The first is to improve the analytic hierarchy process to determine the subjective weight. According to the hierarchical system of Fig 4, according to the calculation steps of the improved analytic hierarchy process in Section 2.1.1, the initial judgment matrices B, B_1_, B_2_, and B_4_ are constructed for the target layer B and the criterion layer X by Eq (1):

$$B=\left(\begin{array}{llll} 1 & 2 & 2 & 2\\ 0 & 1 & 2 & 1\\ 0 & 0 & 1 & 2\\ 0 & 1 & 0 & 1 \end{array}\right),B_{1}=\left(\begin{array}{llll} 1 & 1 & 1 & 0\\ 1 & 1 & 0 & 0\\ 1 & 2 & 1 & 1\\ 2 & 2 & 1 & 1 \end{array}\right),B_{2}=\left(\begin{array}{llll} 1 & 1 & 1 & 0\\ 1 & 1 & 0 & 0\\ 1 & 2 & 1 & 0\\ 2 & 2 & 2 & 1 \end{array}\right),B_{4}=\left(\begin{array}{lll} 1 & 0 & 0\\ 2 & 1 & 0\\ 2 & 2 & 1 \end{array}\right)$$

According to the above initial judgment matrix combination (2) ∼ (9), the subjective weights of the evaluation indexes X_1_ ∼ X_12_ are calculated, as shown in Table 3.

**Table 3:** Weight results of different methods.

Factors	X_1_	X_2_	X_3_	X_4_	X_5_	X_6_	X_7_	X_8_	X_9_	X_10_	X_11_	X_12_
IAHP	0.0372	0.0272	0.0512	0.1107	0.0810	0.0598	0.1480	0.2006	0.1643	0.0126	0.0310	0.0765
EW	0.0207	0.0891	0.0150	0.3097	0.0978	0.0417	0.0489	0.0493	0.0159	0.0845	0.2029	0.0247
CV	0.1134	0.1092	0.0303	0.0407	0.0672	0.1970	0.0826	0.1158	0.0285	0.1528	0.0388	0.0239

The second is the objective weight calculated by the entropy weight method. According to the calculation steps given by the entropy weight method in section 2.1.2, the data is dimensionless by formula (10), and then the difference coefficient is calculated by Eqs (11) and (12), and then the objective weight values of X_1_ ∼ X_11_ are obtained by bringing into formula (13), as shown in Table 3.

Finally, according to the calculation steps of the coefficient of variation in Section 2.1.3, the CV weights of X_1_∼X_11_ are determined by (14) ∼ (17), as shown in Table 3.

The weights determined by the improved hierarchical analysis method, entropy weight method and coefficient of variation method are regarded as linear programming weight intervals, and the constraints of linear programming are constructed according to Eq (18) as shown in Eq (23):

$$\left\{\begin{array}{l} \omega_{1}\in \left[0.0207,0.1134\right],\omega_{2}\in \left[0.0272,0.1092\right]\\ \omega_{3}\in \left[0.0150,0.0512\right],\omega_{4}\in \left[0.0407,0.3097\right]\\ \omega_{5}\in \left[0.0672,0.0978\right],\omega_{6}\in \left[0.0417,0.1970\right]\\ \omega_{7}\in \left[0.0489,0.1480\right],\omega_{8}\in \left[0.0493,0.2006\right]\\ \omega_{9}\in \left[0.0159,0.1643\right],\omega_{10}\in \left[0.0126,0.1528\right]\\ \omega_{11}\in \left[0.0310,0.2029\right],\omega_{12}\in \left[0.0239,0.0765\right] \end{array}\right.$$ (23)

Note: *ω*_1_∼*ω*_12_ are the weights of evaluation indicators X_1_∼X_12_, respectively.

### 4.2 Linear programming optimization weights

In accordance with the theory of linear programming in section 2.2, according to the characteristics of the weights in the evaluation of the collapse hazard, and setting the maximum value of the collapse hazard score as the objective function, this paper establishes the following simplex linear programming standard type function:

$$\max\;Z_{j}=\sum_{j=1}^{12}x_{j}\omega_{j}$$ (24)

$$s.t.\left\{\begin{array}{l} 0.0207\leq \omega_{1}\leq 0.1134,0.0272\leq \omega_{2}\leq 0.1092\\ 0.0150\leq \omega_{3}\leq 0.0512,0.0407\leq \omega_{4}\leq 0.3097\\ 0.0672\leq \omega_{5}\leq 0.0978,0.0417\leq \omega_{6}\leq 0.1970\\ 0.0489\leq \omega_{7}\leq 0.1480,0.0493\leq \omega_{8}\leq 0.2006\\ 0.0159\leq \omega_{9}\leq 0.1643,0.0126\leq \omega_{10}\leq 0.1528\\ 0.0310\leq \omega_{11}\leq 0.2029,0.0239\leq \omega_{12}\leq 0.0765\\ \sum_{j=1}^{11}\omega_{j}=1 \end{array}\right.$$ (25)

After iterative calculations based on the above constraints, specific weight values for the 20 samples were obtained in Table 4.

**Table 4:** Specific weight values of collapse samples.

No.	Weighting of evaluation indicators											
	X_1_	X_2_	X_3_	X_4_	X_5_	X_6_	X_7_	X_8_	X_9_	X_10_	X_11_	X_12_
#01	0.1134	0.1092	0.0512	0.0407	0.0672	0.0417	0.0489	0.0493	0.0462	0.1528	0.2029	0.0765
#02	0.1134	0.0272	0.0512	0.0407	0.0672	0.0417	0.0489	0.0493	0.1643	0.1167	0.2029	0.0765
#03	0.1134	0.0272	0.0512	0.0407	0.0672	0.197	0.0489	0.0493	0.1448	0.1528	0.031	0.0765
#04	0.1134	0.0272	0.0512	0.0407	0.0978	0.0834	0.148	0.0763	0.1226	0.0126	0.2029	0.0239
#05	0.0207	0.0278	0.015	0.0407	0.0978	0.197	0.148	0.0493	0.1643	0.0126	0.2029	0.0239
#06	0.1134	0.1092	0.015	0.0407	0.0672	0.197	0.0489	0.0493	0.099	0.1528	0.031	0.0765
#07	0.1134	0.0272	0.0512	0.0407	0.0672	0.0834	0.148	0.2006	0.1226	0.0126	0.0566	0.0765
#08	0.1134	0.0272	0.0512	0.0407	0.0672	0.197	0.148	0.0709	0.1643	0.0126	0.031	0.0765
#09	0.1134	0.0272	0.0512	0.0407	0.0672	0.197	0.0489	0.17	0.1643	0.0126	0.031	0.0765
#10	0.1134	0.0272	0.0512	0.0407	0.0672	0.197	0.148	0.0709	0.1643	0.0126	0.031	0.0765
#11	0.1134	0.0272	0.0512	0.0407	0.0672	0.1616	0.148	0.2006	0.1226	0.0126	0.031	0.0239
#12	0.1134	0.0272	0.0512	0.0407	0.0888	0.197	0.148	0.0493	0.1643	0.0126	0.031	0.0765
#13	0.1134	0.1092	0.015	0.0407	0.0978	0.0834	0.148	0.0493	0.1226	0.0126	0.1315	0.0765
#14	0.0207	0.0571	0.015	0.3097	0.0672	0.1553	0.148	0.0493	0.0576	0.0126	0.031	0.0765
#15	0.1134	0.1092	0.015	0.0407	0.0978	0.1553	0.148	0.1429	0.0576	0.0126	0.031	0.0765
#16	0.1134	0.1092	0.015	0.3097	0.0978	0.0417	0.0489	0.1283	0.0159	0.0126	0.031	0.0765
#17	0.1134	0.0272	0.015	0.3097	0.0672	0.0417	0.0489	0.2006	0.0159	0.0529	0.031	0.0765
#18	0.1134	0.1092	0.015	0.0407	0.0978	0.1553	0.0489	0.2006	0.099	0.0126	0.031	0.0765
#19	0.0207	0.1092	0.0395	0.0407	0.0672	0.197	0.0489	0.2006	0.0159	0.1528	0.031	0.0765
#20	0.0207	0.1092	0.015	0.0407	0.0672	0.1553	0.0734	0.2006	0.0576	0.1528	0.031	0.0765

### 4.3 Comprehensive evaluation of cloud models

Determine the evaluation rating criteria cloud. Combined with the rank criteria in Table 1, the standard cloud numerical characteristics of each indicator are calculated according to Eq (19), as shown in Table 5.

**Table 5:** Cloud model standard cloud digital characteristics.

Risk	Extreme Danger(Ⅰ)			High Danger(Ⅱ)			Medium Danger(Ⅲ)			Low Danger(Ⅳ)		
Standard cloud parameters	E_xⅠ_	E_nⅠ_	H_eⅠ_	E_xⅡ_	E_nⅡ_	H_eⅡ_	E_xⅢ_	E_nⅢ_	H_eⅢ_	E_xⅣ_	E_nⅣ_	H_eⅣ_
X_1_	175.00	8.33	0.83	125.00	8.33	0.83	75.00	8.33	0.83	25.00	8.33	0.83
X_2_	3.50	0.17	0.02	2.50	0.17	0.02	1.50	0.17	0.02	0.50	0.17	0.02
X_3_	70.00	3.33	0.33	50.00	3.33	0.33	30.00	3.33	0.33	10.00	3.33	0.33
X_4_	3.50	0.17	0.02	2.50	0.17	0.02	1.50	0.17	0.02	0.50	0.17	0.02
X_5_	3.50	0.17	0.02	2.50	0.17	0.02	1.50	0.17	0.02	0.50	0.17	0.02
X_6_	6.00	0.33	0.03	4.00	0.33	0.03	2.00	0.33	0.03	0.50	0.17	0.02
X_7_	3.50	0.17	0.02	2.50	0.17	0.02	1.50	0.17	0.02	0.50	0.17	0.02
X_8_	10.00	3.33	0.33	30.00	3.33	0.33	50.00	3.33	0.33	70.00	3.33	0.33
X_9_	600.00	33.33	3.33	400.00	33.33	3.33	200.00	33.33	3.33	50.00	16.67	1.67
X_10_	5.00	1.67	1.67	20.00	3.33	0.33	40.00	3.33	0.33	60.00	3.33	0.33
X_11_	3.50	0.17	0.02	2.50	0.17	0.02	1.50	0.17	0.02	0.50	0.17	0.02
X_12_	3.50	0.17	0.02	2.50	0.17	0.02	1.50	0.17	0.02	0.50	0.17	0.02

Determine the evaluation cloud. According to Eq (20) and the sample data of collapse risk assessment in Table 2, the digital characteristics of the evaluation cloud are determined, as shown in Table 6.

**Table 6:** Evaluation of cloud digital characteristics.

	X1	X2	X3	X4	X5	X6	X7	X8	X9	X10	X11	X12
Ex	194.95	2.55	48.89	3.70	3.05	1.20	2.60	20.66	483.70	24.25	3.60	3.15
En	103.94	1.57	6.94	0.64	0.95	1.28	1.08	12.46	66.90	19.61	0.70	0.32
He	28.12	0.78	2.00	0.36	0.30	0.55	0.26	4.40	4.58	7.68	0.17	0.18

Determine the comprehensive evaluation cloud. Combined with the weight of each sample index in Table 4, the cloud digital characteristics are evaluated, and the comprehensive cloud digital characteristics are calculated according to the formula (21), as shown in Table 7.

**Table 7:** Comprehensive cloud digital characteristics of each collapse sample.

Evaluating indicator	#01	#02	#03	#04	#05	#06	#07	#08	#09	#10
Expectation Ex	53.46	109.50	100.51	87.60	87.51	76.80	89.72	107.25	109.04	107.25
En entropy value En	4.40	5.14	5.09	4.70	3.85	4.77	4.85	4.99	5.10	4.99
Super entropy He	0.59	0.62	0.68	0.48	0.19	0.63	0.63	0.54	0.64	0.54
Evaluating indicator	#11	#12	#13	#14	#15	#16	#17	#18	#19	#20
Expectation Ex	89.55	106.87	85.39	36.37	55.61	35.74	37.91	76.57	22.86	41.84
En entropy value En	4.86	4.96	4.65	2.78	4.30	3.93	4.12	4.67	3.10	3.49
Super entropy He	0.64	0.53	0.47	0.10	0.50	0.49	0.60	0.61	0.41	0.41

Determine the comprehensive level risk cloud. Combined with the calculation results of the standard cloud digital characteristics in Table 5, the comprehensive level risk cloud digital characteristics are calculated according to formula (22), as shown in Table 8.

**Table 8:** Comprehensive grade risk cloud digital characteristics.

Risk classification	Extreme Danger(Ⅰ)			High Danger(Ⅱ)			Medium Danger(Ⅲ)			Low Danger(Ⅳ)		
Comprehensive grade risk cloud parameters	E_xⅠ_	E_nⅠ_	H_eⅠ_	E_xⅡ_	E_nⅡ_	H_eⅡ_	E_xⅢ_	E_nⅢ_	H_eⅢ_	E_xⅣ_	E_nⅣ_	H_eⅣ_
	85.42	4.97	0.58	61.48	5.06	0.51	37.81	5.06	0.51	19.42	3.30	0.33

**Fig 5:**
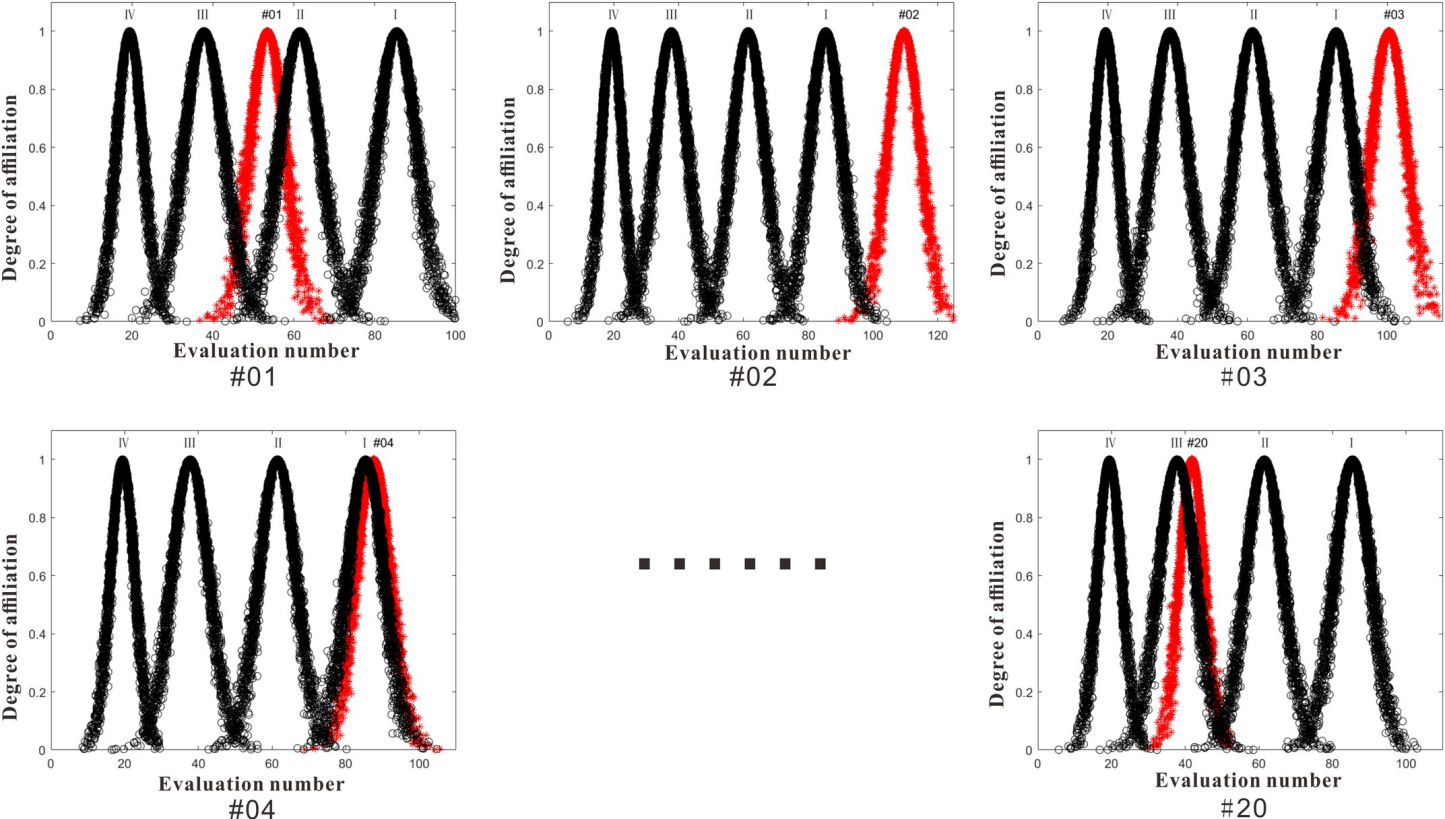
Risk comparison chart of each sample.

Determine the risk level. Using Matlab2021a, the integrated cloud digital features of the above collapse samples and the integrated grade risk cloud digital features, were plotted in the same spatial coordinate system to determine the risk level of each collapse, and the risk comparison diagram of each evaluation sample is shown in Fig 5, and the final evaluation results of each sample are shown in Table 9.

**Table 9:** Comparison of evaluation results of each method.

No.	Evaluation results of this paper	entropy weight method	Fuzzy Integrated Evaluation Method	EW-AHP evaluation results	Results of on-site investigations
#01	Ⅱ	Ⅱ	Ⅱ	Ⅱ	collapse
#02	Ⅰ	Ⅱ	Ⅱ	Ⅱ	collapse
#03	Ⅰ	Ⅱ	Ⅰ	Ⅰ	collapse
#04	Ⅰ	Ⅰ	Ⅱ	Ⅱ	collapse
#05	Ⅰ	Ⅱ	Ⅱ	Ⅱ	collapse
#06	Ⅰ	Ⅱ	Ⅱ	Ⅱ	collapse
#07	Ⅰ	Ⅰ	Ⅰ	Ⅰ	collapse
#08	Ⅰ	Ⅰ	Ⅱ	Ⅰ	collapse
#09	Ⅰ	Ⅰ	Ⅱ	Ⅱ	collapse
#10	Ⅰ	Ⅰ	Ⅱ	Ⅰ	collapse
#11	Ⅰ	Ⅰ	Ⅲ	Ⅰ	collapse
#12	Ⅰ	Ⅰ	Ⅱ	Ⅱ	collapse
#13	Ⅰ	Ⅱ	Ⅱ	Ⅱ	collapse
#14	Ⅲ	Ⅲ	Ⅱ	Ⅱ	collapse
#15	Ⅱ	Ⅱ	Ⅱ	Ⅱ	collapse
#16	Ⅲ	Ⅲ	Ⅱ	Ⅱ	collapse
#17	Ⅲ	Ⅱ	Ⅱ	Ⅱ	collapse
#18	Ⅰ	Ⅰ	Ⅱ	Ⅱ	collapse
#19	Ⅳ	Ⅱ	Ⅱ	Ⅱ	collapse
#20	Ⅲ	Ⅱ	Ⅱ	Ⅱ	collapse

As can be seen from Table 9 and 13 of the evaluation results of this paper’s method are extremely hazardous, 2 are highly hazardous, 4 are moderately hazardous, and 1 is low hazardous, in which the extremely hazardous situations reach 65% of the total number of collapses; according to the results evaluated by entropy weight method, it can be seen that there are only 8 extremely hazardous situations, which account for 40% of the total number of collapses, and the evaluation results of fuzzy comprehensive evaluation method and EW-AHP method are poorer overall, thus it can be seen that the improved combined assignment method-cloud model proposed in this paper is more accurate, which verifies the reasonable evaluation results of linear programming-variable weight cloud modeling method. The results are poorer, which shows that the improved combined assignment method-cloud model proposed in this paper is more accurate, and the evaluation results are closer to the actual situation, which verifies the reasonableness of the evaluation results of the linear programming-variable weight cloud modeling method. The method is characterized in detail in the following discussion section.

## 5 Discussion

From it can be seen from the above analysis of the results, the evaluation results of this paper’s method are better than other evaluation methods, this is due to the existence of different evaluation methods with different advantages and disadvantages, for example: the entropy weight method is more objective when carrying out the process of assigning the weights of the indexes, but it is more dependent on the data, and from the fact that most of the basic data of the weathering degree of rock (X11) in Table 2 are skewed towards the extreme danger as well as the fact that in Table 3, the index is given a large weight (0.2029) can verify this, so the entropy weighting method weights cannot be used as the actual importance of the indicator, which will make the evaluation results have some bias; as for the fuzzy comprehensive evaluation method, it has good applicability in dealing with the problem of hierarchical affiliation of various uncertainty indicators, but it cannot take into account the randomness of the sample data, which will lead to poor evaluation results; the EW-AHP method has the advantage of synthesizing the subjective and objective weights and is widely used in many fields, but it is more reliant on the comprehensive evaluation method, and it is also limited by the way of weight coupling of the two methods.

The linear programming variable weight-cloud model method in this paper is better than these methods in dealing with the problems encountered by the above methods. Firstly, the linear programming method in this paper, when calculating the weights, is not carried out for all the data in the samples only, but utilizes the method of assigning weights like in Table 3 to determine a sample interval, which not only integrates the advantages of the weighting methods, but also avoids the limitations of the process of combining the weights. Secondly, in the weight assignment process, the weight of the indicators calculated by the linear programming method is not a fixed value (as in Table 4), but is based on the basic data of different indicators in the samples to carry out the corresponding indicator weight variation, which fully takes into account the randomness of the data; finally, the cloud model evaluation method is used to classify the indicators, which can take into account the randomness of the data and the indicator grading standard at the same time, and the evaluation results are simple and concise. The method can simultaneously take into account the randomness of data and the fuzziness of the index grading standard, and the evaluation results are concise and intuitive. Therefore, the application of linear programming variable weighting-cloud modeling method in the evaluation of collapse risk is reasonable and feasible.

In order to verify whether the methods of this paper can still maintain its efficient performance when encountering a variety of different data inputs, eight sets of data from the literature [17] are introduced for quantification to verify the robustness of the evaluation method (see Table 10 for the base data). The evaluation results are shown in Table 11.

**Table 10:** Validation data sheet.

Sample number	Evaluating indicator											
	X_1_	X_2_	X_3_	X_4_	X_5_	X_6_	X_7_	X_8_	X_9_	X_10_	X_11_	X_12_
BT05	73	4	70	1	3	3	4	1	1253	6	3	2
BT09	613	2	50	4	3	2	2	1	1253	18	3	2
BT13	105	4	69	3	2	4	3	1	1253	20	3	2
BT24	234	3	30	2	3	2	3	1	1253	25	3	2
BT33	119	2	48	1	2	2	1	1	1253	25	3	2
BT40	21	4	85	1	2	1	2	1	1253	35	3	2
BT49	189	1	70	2	3	4	3	1	1253	8	3	2
BT05	73	4	70	1	3	3	4	1	1253	6	3	2

**Table 11:** Validation results table.

Sample number	BT05	BT09	BT13	BT24	BT33	BT40	BT49	BT54
Evaluation results of the methods in this paper	Ⅰ	Ⅰ	Ⅱ	Ⅰ	Ⅰ	Ⅱ	Ⅱ	Ⅱ
Evaluation results in the literature [17]	Ⅱ	Ⅲ	Ⅱ	Ⅲ	Ⅲ	Ⅲ	Ⅱ	Ⅲ

The results showed that samples BT05, BT09, BT24, and BT33 were evaluated as being extremely hazardous (I), and BT13, BT40, BT49, and BT54 were evaluated as being highly hazardous (II), which is higher than that of the samples in the literature [17], which is in line with the concept of the prevention and control perspective in this paper. This has also demonstrated that linear programming remains efficient in finding the most hazardous score with optimal weight assignment when encountering a variety of different data inputs, validating the robustness of the model.

Although this paper verifies the reasonableness and feasibility of the proposed method after comparative analysis and actual data, there are still some limitations: in the final evaluation result of this paper’s method, the evaluation result of the sample numbered #19 is Ⅳ, which according to the evaluation result is the most unlikely sample point to collapse, but in reality, it collapsed, and we analyze the reasons for this from the principle of the method: in order to fully combine the advantages of the different weighting methods advantages, the weights are determined in the convergence interval with a certain degree of ambiguity; at the same time, linear programming will be assigned according to the basic data when weights are assigned, and the cloud model will take into account the data of the samples as well as the weights of the samples when comprehensive cloud processing is carried out to compute the final cloud parameters (see Table 7), whereas in the real situation, the data is not collected perfectly, and there are often some defects, which will make the assignment of the weights more extreme and calculate the cloud parameters with large errors, leading to some bias in the evaluation results. Overall, the evaluation of collapse hazard based on linear programming variable weighting-cloud model is in line with practical engineering. In the future, we will try to use linear programming combined with geographic information system (GIS) to study the regional collapse hazard situation using the allocation principle of linear programming to better visualize the degree of collapse hazard.

## 6 Conclusion

(1) This paper proposes a collapse risk assessment method based on improved combination weighting-cloud model. Linear programming is used as the weight combination method, and the index weights are assigned according to the basic data of each sample. Combined with the comprehensive evaluation method of cloud model, the evaluation indexes are transformed into corresponding digital features, and the fuzziness and randomness of data and indexes are considered.
(2) This paper calculates the weight of each sample index from the perspective of prevention and control. By taking the score at the most dangerous time of collapse as the objective function and the weight as the decision variable, the index weight calculated according to the basic data is the specific weight of each collapse sample, which is conducive to taking targeted prevention and control measures according to the different emphasis of different collapse point weight values.
(3) The evaluation results based on the improved combination weighting-cloud model method have a reasonable error interval compared with other methods, but the method in this paper is more in line with the actual situation of the study area. Through the comparison of comprehensive evaluation results, it is proved that the method proposed in this paper is reasonable and feasible, which has scientific guiding significance for the prevention and control of collapse in this type of area.

## Supporting information

S1 File (ZIP)
